# Role of Common Fractalkine Receptor Variants with Chronic Hepatitis B Patients in Tunisia

**DOI:** 10.3390/v17070968

**Published:** 2025-07-10

**Authors:** Imene Ben Dhifallah, Kaouther Ayouni, Zeineb Belaiba, Bacem AlaDdine Razgui, Sahar Trabelsi, Henda Touzi, Amel Sadraoui, Walid Hammemi, Hela Hannachi, Amira Kebir, Slimane Ben Miled, Henda Triki

**Affiliations:** 1 Laboratory of Clinical Virology, Pasteur Institute of Tunis, University of Tunis El Manar, 13 Place Pasteur, BP 74, Tunis 1002, Tunisia; kaouther.ayouni@gmail.com (K.A.); zeineb.belaiba114@gmail.com (Z.B.); touzihenda@yahoo.fr (H.T.); amelsedraoui1@gmail.com (A.S.); hammemi.walid12@gmail.com (W.H.); helahanrejeb@gmail.com (H.H.); henda.triki@pasteur.tn (H.T.); 2 Research Laboratory: “Virus, Vector and Host” (LR20IPT02), Pasteur Institute of Tunis, University of Tunis El Manar, 13 Place Pasteur, BP 74, Tunis 1002, Tunisia; 3 Faculty of Sciences of Tunis, University of Tunis El Manar, Campus Universitaire El Manar, Tunis 2092, Tunisia; 4 Laboratory of Bioinformatic, Biomathematics and Biostatistics, Pasteur Institute of Tunis, University of Tunis El Manar, 13 Place Pasteur, BP 74, Tunis 1002, Tunisia; becem.rezgui@etudiant-fst.utm.tn (B.A.R.); sahartrabelsi91@gmail.com (S.T.); amira.kebir@pasteur.utm.tn (A.K.); slimane.benmiled@gmail.com (S.B.M.); 5 Faculty of Medicine of Tunis, University of Tunis El Manar, 15 Rue Djebel Lakhdhar, La Rabta, Tunis 1007, Tunisia

**Keywords:** CHB, PCR, CX3CR1, fractalkine receptor, polymorphism, FDR

## Abstract

Chronic hepatitis B virus (CHB) infection remains a leading cause of hepatic inflammation and damage. Several studies have suggested the significant role of CX3C chemokine receptor 1 (CX3CR1) in inflammatory damages. The polymorphisms V249I and T280M affect receptor expression and function. In the current study, we investigated the association of V249I and T280M variants of the CX3CR1 fractalkine receptor with susceptibility to CHB disease. In total, 280 patients with chronic hepatitis B and 260 controls from different cities of Tunisia recruited in the Pasteur Institute of Tunisia between January 2017 and December 2022 were genotyped for the V249I and T280M CX3CR1 gene. The allele and genotype frequencies of these variants did not show significant associations with susceptibility to CHB infection (*p* > 0.05). Analysis of allele and genotype frequencies showed that there was no differences in age and sex distribution between patients and the control group, but when CHB patients were stratified according to age, a clear significant difference was obtained for the T280M polymorphism (*p* < 10^−3^, OR = 88.91; *p* < 10^−3^, OR = 37.42, for genotype and allelic distribution, respectively) with the MM genotype being more frequent in patients aged ≥ 50 years. The most frequently combined genotypes in the Tunisian population were VVTT, VITT and VITM both in patients (48.9%, 22.5% and 22.1%, respectively) and in controls (52%, 23.8%, 13.5%, respectively) compared to the extremely rare IITT, IITM or IIMM genotypes. In conclusion, this study suggests a noteworthy genotype–age association, particularly involving the T280M variant

## 1. Introduction

According to the World Health Organization, an estimated 254 million people were chronically infected with the hepatitis B virus (HBV) in 2022, and HBV was responsible for approximately 1.1 million deaths globally, mostly due to cirrhosis and hepatocellular carcinoma. Although both viral and host parameters are implicated in the pathogenesis of HBV infection, the hepatocyte damage associated with HBV infection is believed to be mainly caused by immune-mediated mechanisms. A robust T-cell response is crucial for the spontaneous clearance of HBV infection, while the host’s inability to mount an effective immune response has been linked to viral persistence [1,2,3]. Notably, cytokines play a key role in suppressing viral expression and replication, thereby controlling the infection without inducing the death of infected cells. The progression to chronic infection is associated with a weak or undetectable humoral immune response, characterized by the persistent presence of hepatitis B surface antigen (HBsAg) in the bloodstream [4,5]. Chemokines can be classified into four subgroups as CXC-, CC-, C-, and CX3C depending on the position of the first two cysteine residues [6]. CX3CR1 (Fractalkine-CX3CL1-receptor) gene, also known as fractalkine receptor (OMIM: 601470), is expressed in a variety of inflammatory cells in the brain and eye, including neutrophils, monocytes, microglia, T lymphocytes, and solid organs [7]. It was involved in several inflammatory processes via induction of leukocyte recruitment to the site of inflammation [8,9]. This chemokine–receptor system has a major pain-modulatory effect, with a predominantly central pro-algesic and a peripheral anti-algesic component [10]. Interestingly, in addition to its chemotactic activity, fractalkine has unprecedented adhesion-mediating properties [11]. When anchored to the membrane, it may play an important role in the immune response by reinforcing cell contact. To investigate whether CX3CR1 may regulate chronic hepatitis B virus (CHB) disease, we aimed to study genetic variants associated with susceptibility to chronic hepatitis B infection. There are two common nonsynonymous single-nucleotide polymorphisms in the CX3CR1 gene, which result in the V249I (rs3732379: G > A) and T280M (rs3732378: C > T) substitutions. These nonsynonymous SNPs are functionally relevant as they influence the binding of fractalkine to CX3CR1 [12]. Specifically, the 249I and the 280M alleles result in fewer receptor binding sites and decreased ligand affinity [13,14].

The I249 and M280 alleles have been widely associated with various inflammatory diseases, including atherosclerosis, coronary artery disease, internal carotid artery (ICA) occlusion, and cerebral infarction, as demonstrated by numerous studies conducted in the Caucasian population [12,13,15,16,17,18]. These studies have suggested the significant role of CX3CR1 in inflammatory conditions, yet, to our knowledge, there are no data and published studies regarding the role of CX3CR1 nonsynonymous SNPs with respect to CHB. The aims of this study were to examine the role of CX3CR1 gene variants in Tunisian chronic hepatitis B patients and to analyze the correlations between CX3CR1 polymorphisms and the demographic and clinical features of the patients.

## 2. Materials and Methods

### 2.1. Study Population

The patient group included 280 chronic HBV carriers (HBsAg and anti-HBc positives two times at least 6 months apart), referred to the Laboratory of Virology in Pasteur Institute (Tunisia) between January 2017 and December 2022 for HBV viral load, as part of a pretherapeutic investigation. The viral load analyzed in our study corresponds to baseline measurements before the initiation of any antiviral treatment. The patient group consisted of Caucasians, primarily of Arab and Berber descent, originating from different cities in Tunisia. The study included 131 males aged between 18 and 86 years (mean = 47.26 years) and 149 females aged between 26 and 86 years (mean = 44.34 years). HBV DNA levels were measured using the High-Pure COBAS TaqMan commercial Kit from Roche Molecular Systems, according to the manufacturer’s instructions. Patients were divided into two groups according to their viral load, with an arbitrary threshold value of 2000 IU/mL. Group #1 included patients with a viral load over 2000 IU/mL (N = 43) and Group #2 included patients with a viral load below 2000 IU/mL (N = 237). The threshold 2000 IU/mL was proposed by the Tunisian Society of Gastroenterology and the National Institutes of Health Conference to classify patients as inactive HBV carriers or active replicating carriers. The control group was composed of 260 healthy individuals who came to the Institute for routine laboratory investigations. A questionnaire was used to exclude patients with a known hepatitis history or with symptoms of possible ongoing infection. Lastly, 5 mL of venous blood with ethylenediaminetetraacetic acid (EDTA) was collected from each patient and control.

### 2.2. Genotyping

Genomic DNA was extracted from EDTA anticoagulated whole blood using the conventional salting-out procedure. Polymerase chain reaction–restriction fragment length polymorphisms (PCR-RFLP), as previously described [19], and direct sequencing methods were used for determining genetic variations. The region containing the polymorphic sites V249I and T280M was amplified with the following set of primers: forward 5′-CCGAGGTCCTTCAGGAAATCT-3′ and backward 5′-TCAGCATCAGGTTCAGGAACTC-3′. The resulting PCR product (588 bp) was used for RFLP analysis [20]. The restriction enzyme Ac1I (New England BioLabs) was used for the genotyping of the V249I polymorphism. Mutated homozygotes I/I249 were represented by an undigested PCR 588 bp product; mutated heterozygotes V/I249 were represented by fragments 588 + 383 + 205 bp; and the wild-type homozygotes V/V249 were represented by fragments 383 + 205 bp. The restriction enzyme BsmBI (New England BioLabs, Ipswich, MA, USA) was used for the genotyping of the T280M polymorphism. Mutated homozygotes M/M280 were represented by fragments 372 + 216 bp; mutated heterozygotes T/M280 were represented by fragments 372 + 297 + 216 + 75 bp; and wild-type homozygotes T/T280 were represented by fragments 297 + 216 + 75 bp. All fragments were separated on 3% agarose gels and visualized with UV light after ethidium bromide staining. To validate PCR-RFLP genotyping results, 5% of samples for each genotype were reanalyzed using Sanger sequencing. Sequencing was performed on an ABI Prism 3130-Genetic Analyzer using forward PCR primer as the sequencing primer, purified PCR-products (purified using the QIAquick PCR purification Kit, QIAGEN, Hilden, Germany), and the Big Dye terminator ready reaction cycle sequencing kit (Applied Biosystems, Waltham, MA, USA). Nucleotide sequence analysis yielded results consistent with the PCR-RFLP method.

### 2.3. Statistical Analysis

Differences in allele and genotype frequencies between patients and healthy controls were compared using the standard chi-squared test (Epistat statistical package). When the expected cell number was less than 5, Fisher’s exact test was used. The logistic regression model was also employed to assess the association between the genetic variants V249I and T280M and the likelihood of being a patient with HBV, as well as to account for age and gender. Four genetic models were used (recessive, dominant, over-dominant, and additive) in this analysis. In the patient group, analysis was conducted according to gender, sex, and viral load. Probability values of 0.05 or less were regarded as statistically significant. The strength of a gene association is indicated by the odds ratio (OR). The OR and the 95% confidence intervals (CI) were calculated whenever applicable. To account for the increased risk of type I error due to multiple testing, the Benjamini–Hochberg False Discovery Rate (FDR) correction was applied whenever appropriate. Statistical significance was defined as FDR-adjusted *p* < 0.05.

## 3. Results

Table 1 shows the demographic data and the viral load range for the studied population. The 280 patients and 260 controls were well matched in terms of age and gender.

Table 2 shows the allele and genotype frequencies in the two groups. The distributions of V249I and T280M genotypes were in Hardy–Weinberg equilibrium for patients and controls. Analysis of allele and genotype frequencies showed that the allele and genotype frequencies of CX3CR1 polymorphisms (T280M and V249I) did not show significant associations with susceptibility to CHB infection (*p* > 0.05), with M allele, MM genotype and I allele, II genotype were rare for the T280M and V249I polymorphisms, respectively, in the Tunisian population. Table 3 shows the haplotypic frequency of each study group; none of the subjects were found with VVTM, VVMM, and VIMM genotypes. The most frequent combined genotypes in the Tunisian population were VVTT, VITT, and VITM, both in patients (48.9%, 22.5%, and 22.1%, respectively) and in controls (52%, 23.8%, and 13.5%, respectively) compared to the extremely rare IITT, IITM, or IIMM genotype. Analysis of allele and genotype frequencies showed that there was no statistical difference in age and sex distribution between patients and the control group; however, the risk increases with age, with borderline significance for T280M and V249I (Table 4). When analyses of the genotype and allelic distribution were performed in patients according to HBV viral load, an insignificant difference was found between the two patient subgroups for the two nonsynonymous SNPs (*p* = 0.67 and *p* = 0.75 for genotype distribution, and *p* = 0.74 and *p* = 0.65 for allelic distribution, for the T280M and V249I polymorphisms, respectively). However, when CHB patients were stratified according to age, a clear significant difference was obtained for the T280M polymorphism (*p* < 10^−3^, OR= 88.91; *p* < 10^−3^, OR = 37.42, for genotype and allelic distribution, respectively) with the MM genotype being more frequent in patients aged ≥ 50 years. The V249I polymorphism showed no significant differences in allele or genotype frequencies between those two groups (Table 5). The same age–genotype relationship was not observed in the control group. No significant association was found between the genotype of healthy controls stratified by age and the two nonsynonymous SNPs (*p* = 0.39 for the T280M polymorphism and *p* = 0.20 for the V249I polymorphism). In addition, we did not detect a significant correlation between allele and participation age in controls for the T280M polymorphism (*p* = 0.27) and for the V249I polymorphism (*p* = 0.10).

## 4. Discussion

CX3CR1 is a receptor for the chemokine CX3CL1 (fractalkine), which plays a crucial role in recruiting immune cells to sites of inflammation, including those infected with HBV. CX3CL1 expression levels differ among HBV genotypes, and its expression in HBV-replicating hepatocytes and hepatoma cells may contribute to the immunopathogenesis of HBV infection [21]. The interaction between CX3CL1 and CX3CR1 can also influence the immune responses against HBV infection. For example, the frequency of PD-1 (high) CX3CR1(+) CD8(+) T cells in HCC with HBV infection is significantly higher than in HCC with hepatitis C virus (HCV) infection [22]. CX3CR1 SNPs can modulate both the concentration and biological activity of CX3CR1 and CX3CL1 in the body, affecting the complex aforementioned patterns of virus and host responses during chronic HBV infections, as well as how they interact with each other. These changes can impact immune cell activity and overall liver health, which are important in the context of HBV [21,22,23,24]. Furthermore, CX3CR1 SNPs are linked to hepatitis B virus (HBV) infection and disease progression, potentially influencing HBV viral load. Studies have shown associations between specific CX3CR1 SNPs and persistent HBV infection, viral load, and even the development of liver fibrosis [25]. For instance, rs477515 and rs7756516 have been associated with viral load in a study involving a cohort of individuals with hepatocellular carcinoma (HCC) and HBV [26]. This study aimed to investigate the association between CX3CR1 polymorphisms (T280M and V249I), age, sex, and CHB disease risk using different genetic models (recessive, dominant, over-dominant, and additive).

Our results showed that no significant associations were observed between allele and genotype frequencies between patients with CHB and control subjects. Previous studies have reported limited or non-significant effects on HBV susceptibility [20]. Mühlbauer et al. found no association between V249I CX3CR1 and liver disease, especially with hepatocellular carcinoma [20]. Additionally, no association was found when the presence of extrahepatic metastasis or the degree of fibrosis in non-tumorous tissue was considered. The lack of significant differences in allele/genotype frequencies is consistent with research suggesting that T280M and V249I polymorphisms are rare and may not play a central role in HBV pathogenesis. Conversely, the 249I allele was strongly associated with advanced liver fibrosis, and this disease was associated with carrying at least one copy of the 249I allele [21]. According to recent studies, the fractalkine-CX3CR1 axis is critical both in the diagnosis and in the prognosis of HCC, because it can not only regulate the immune response, but can also regulate the cell cycle of HCC [27]. As a matter of fact, relevant experiments indicate that fractalkine can enhance the anti-tumor effect of the immune system against HCC in mice: Tang et al. observed that CX3CL1 can elicit tumor-specific cytotoxic T cells and an increased production of IL-2 and IFN-γ capable of inhibiting tumor growth [28]. With specific concern on HBV chronic infection, it was demonstrated that a differential expression of CX3CL1 produced by HBV-infected hepatocytes and hepatoma cells can affect the migration activity of CX3CR1+ immune cells, potentially contributing to the immunopathogenesis of HBV infection [22]. This in vitro evidence was confirmed in vivo by Matsubara et al. who found that cirrhotic hepatitis B HCC patients who have a high expression of fractalkine and its receptor CX3CR1 have also a low rate of recurrence of intrahepatic or extrahepatic metastases, which suggest that their expression may be related to the prognosis of HCC patients, and may be involved in tumor immunity by killing tumor cells [27]. However, some researchers have proposed that there may be a lack of association between the CX3CL1–CX3CR1 axis and HCC, as studies have demonstrated that CX3CR1 is not a risk factor for HCC [20]. These researchers believe that tumor-derived chemokines have dual roles. Leukocyte aggregation following a signal of increasing chemokine concentration may not only be beneficial to the host but also contribute to tumor growth. Therefore, the roles played in tumors by virus-related chemokines may be multiple, and the specific role of CX3CR1 in the formation of chronic hepatitis and liver disease still needs to be further elaborated [29].

Many other reports found an association of polymorphisms in CX3CR1 and other diseases such as coronary artery disease [13,30], risk of acute rejection in renal transplant recipients [31], cancer rates in renal transplant recipients [32], age-related macular degeneration [33], and HIV [14]. Wu et al. showed that the CX3CR1 T280M and V249I polymorphisms are associated with susceptibility to atherosclerosis (AS) [34]. Authors suggested that the CX3CR1 280M and 249I allele carriers had atheroprotective roles on AS in the heterozygote state, and the 280M allele carriers were associated with susceptibility to AS in the homozygote state [34]. Cabanski et al. analyzed the association between T280M polymorphisms and acute tonsillitis and suggested that T280M polymorphism could be associated with a reduced risk of the disease and that T280M (rs3732378: C > T) substitutions were found to be higher in controls than in the patients [35]. Some other studies on HIV infection or colorectal cancer found that the CX3CR1 T280M and V249I polymorphisms are not associated with susceptibility to disease [19,36]. The various aetiopathogeneses of the inflammatory disease, genetic backgrounds, and different environmental factors, such as different exposures in different populations might have caused the discrepant results. According to reported studies, the distribution of the two common nonsynonymous SNPs in the CX3CR1 gene, V249I and T280M, varies among different ethnic groups [37,38,39,40]. In the study by Li et al., it was demonstrated that the allelic frequencies of these polymorphisms were higher in Caucasian populations compared to Asian and West African populations, where the I249 and M280 alleles were either absent or present at very low frequencies [40]. Ethnic differences in CX3CR1-related disease susceptibility were also highlighted in an Algerian population, where the T280M was associated with an increased risk of neovascular age-related macular degeneration, whereas no such association was observed in Greek or Indian populations [37,38,39]. However, in the Greek population, it was the V249I variant which was linked to an increased risk of geographic atrophy a form of age-related macular degeneration [38].

In a previous Tunisian study, authors found no association between T280M and V249I polymorphisms and asthma disease [41]. Distributions in the control population showed somewhat similar results when the proportions were compared between the control populations in their study and those in our study. (VV: 56%, VI: 37%, and II: 6%) vs. (VV: 52%, VI: 37.3% and II: 10.7%) and (TT: 85%, TM: 14%, MM: 1%) vs. (TT: 79.6% TM: 18.9%, MM: 1.5%), respectively for the V249I and the T280M polymorphisms.

In this study, we also investigated the association between CX3CR1 polymorphisms (T280M and V249I), demographic data, and HBV viral load in patients. No significant association was found when patients were stratified by viral load or gender. However, when stratified by age, a statistically significant difference was observed for the T280M polymorphism. Specifically, the MM genotype was significantly more frequent among older CHB patients. This genotype–age association was not observed in the control group. This finding may suggest an enrichment of the MM genotype among older individuals with chronic HBV infection, possibly due to age-related factors affecting immune regulation, disease progression, or genotype-specific susceptibility. It may also indicate a differential role of CX3CR1-mediated immune surveillance across the lifespan. The T280M variant, which results in a threonine-to-methionine substitution, has been associated with an altered CX3CR1 receptor function and reduced binding affinity to its ligand, fractalkine (CX3CL1) [42]. This alteration may impair the recruitment of cytotoxic T lymphocytes and natural killer (NK) cells to infected hepatocytes, thereby contributing to delayed immune-mediated clearance of HBV. Over time, carriers of the M allele may exhibit prolonged immune tolerance or low-grade inflammation, allowing for persistent viral replication and delayed progression to clinically apparent disease. To date, there is limited evidence in the literature regarding the relationship between aging and CX3CR1 gene polymorphisms (V249I and T280M) in CHB patients. Further studies are warranted to explore this interaction, particularly in light of the fact that older individuals tend to develop more severe liver damage and fibrosis. The potential contribution of CX3CR1 to liver inflammation and fibrosis in chronic liver disease may be influenced by age-related immune decline, which progressively affects various physiological systems. However, the underlying biological mechanisms remain unclear. One limitation of our study is the lack of data on the age at HBV infection, which prevents us from determining the duration of infection. Future prospective studies are necessary to address this issue. Additionally, none of the patients in our cohort had a current or past diagnosis of HBV-related hepatocellular carcinoma (HCC), as this was not part of the inclusion criteria. Further large-scale studies are needed to investigate the association between CX3CR1 polymorphisms and HCC development, comorbidities related to chronic liver disease, and the stage of hepatic fibrosis at baseline.

## 5. Conclusions

To the best of our knowledge, this is the first study reporting the association between the polymorphism of CX3CR1 and chronic hepatitis B. A noteworthy genotype–age association was observed, particularly involving the T280M variant, suggesting a possible role of age-related immune mechanisms in modulating HBV persistence through CX3CR1-related pathways. These findings emphasize the need for further research to elucidate the underlying immunogenetic mechanisms linking CX3CR1 polymorphisms to HBV chronicity and to evaluate their potential implications for prognosis or targeted therapeutic approaches. The study ensured quality control by verifying genotypes through sequencing in a substantial subset of samples, confirming the reliability of the results. However, some limitations of this study should be acknowledged, and other studies targeting larger sample sizes and other ethnic populations are warranted to further assess the impact of these polymorphisms on the chronicity of hepatitis B disease. The studied polymorphisms are relatively rare in the Tunisian population, which may reduce the likelihood of finding statistically significant associations in the general population. The sample sizes in some subgroups of the CX3CR1 T280M and V2491 polymorphisms were relatively small, not having enough statistical power to explore the real association.

## Figures and Tables

**Table 1:** Demographic data and HBV viral load in patients and controls.

	HBV Patients (%)	Controls (%)
	N = 280	N = 260
Mean Age (year)	45.3	41.1
Gender		
Females	149 (53.2)	143(55.0)
Males	131 (46.8)	117 (45.0)
Viral load		
<2000 IU/mL (group #1)	237 (84.6)	-
>2000 IU/mL (group #2)	43 (15.4)	-
Viral load Median [IQR]	113.5 [9–874.2]	-
HBV Serology		
HBsAg	Positive	-
Anti-HBc	Positive	-

IU corresponds to 5.6 viral copies.

**Table 2:** Allele and genotype frequencies of T280M and V249I polymorphisms among patients with CHB and control subjects.

Amino Acid	Patients (N = 280) N(%)	Controls (N= 260) N(%)	χ^2^	OR	95% IC	*p*-Value	Additive Model		Overdominant Model		Dominant Model		Recessive Model	
							OR CI 95%	P adj	OR CI 95%	P adj	OR CI 95%	P adj	OR CI 95%	P adj
T280M														
T/T	209 (74.6)	207 (79.6)	4.78	0.75	0.50–1.13	0.09	0.82	0.35	1.44	0.22	3.95	0.32	0.75	0.39
T/M	70 (25.0)	49 (18.9)		1.44	0.95–2.17		0.56–1.19		0.95–2.18		0.44–35.69		0.50–1.12	
M/M	1 (0.4)	4 (1.5)		0.23	0.03–2.07									
T	488 (87.1)	463 (89.0)	0.92	0.83	0.58–1.21	0.34								
M	72(12.9)	57(11.0)												
V249I														
V/V	137 (49.0)	135 (52.0)	4.99	0.89	0.63–1.24	0.08	1.02	0.89	1.37	0.22	1.73	0.22	0.87	0.49
V/I	125 (44.6)	97 (37.3)		1.36	0.96–1.91		0.78–1.33		0.97–1.94		0.93–3.23		0.62–1.22	
I/I	18 (6.4)	28 (10.7)		0.57	0.31–1.06									
V	399 (71.3)	367 (70.6)	0.06	1.03	0.79–1.34	0.81								
I	161 (28.7)	153 (29.4)												

Abbreviations: OR: Odds ratio; 95% IC: 95% confidence interval; N: total number of subjects. P adj: *p*-values adjusted using Benjamini–Hochberg FDR correction. *p*-value: significant *p*-value (*p*-value < 0.05).

**Table 3:** Combined genotype frequencies of the CX3CR1 V249I and T280M in CHB patients and in controls.

**Combined Genotype**	**CX3CR1**		**Patients**	**Controls**
	**V249I**	**T280M**	**(N = 280)** **N (%)**	**(N = 260)** **N (%)**
1	VV	TT	137 (48.9)	135 (52.0)
2	VV	TM	0 (0.0)	0 (0.0)
3	VV	MM	0 (0.0)	0 (0.0)
4	VI	TT	63 (22.5)	62 (23.8)
5	VI	TM	62 (22.1)	35 (13.5)
6	VI	MM	0 (0.0)	0 (0.0)
7	II	TT	9 (3.2)	10 (3.8)
8	II	TM	8 (2.9)	14 (5.4)
9	II	MM	1 (0.4)	4 (1.5)

**Table 4:** Multivariable logistic regression analysis of T280M and V249I polymorphism distribution by gender and age in patients with CHB and control subjects.

	Additive Model			Over-Dominant Model			Dominant Model			Recessive Model		
	OR	CI 95%	P adj	OR	CI 95%	P adj	OR	CI 95%	P adj	OR	CI 95%	P adj
T280M												
Gender	1.03	0.73–1.45	0.9	1.02	0.73–1.44	0.9	1.05	0.74–1.48	0.9	1.02	0.73–1.44	0.9
Age	1.43	0.99–2.07	0.15	1.43	0.99−2.06	0.15	1.39	0.97–2.01	0.15	1.43	0.99–2.07	0.15
V249I												
Gender	1.04	0.74–1.47	0.9	1.04	0.74–1.47	0.9	1.06	0.75–1.49	0.9	1.04	0.74–1.46	0.9
Age	1.41	0.98–2.04	0.15	1.44	0.99–2.07	0.15	1.4	0.97–2.02	0.15	1.43	0.99–2.06	0.15

Abbreviations: OR: Odds ratio; 95% IC: 95% confidence interval; N: total number of subjects. P adj: *p*-values adjusted using Benjamini–Hochberg FDR correction. *p*-value: significant *p*-value (*p*-value < 0.05).

**Table 5:** Distribution of allele and genotypic frequencies of *T280M* and *V249I* polymorphisms among patients with CHB according to age groups.

Amino Acid	Age		χ^2^	OR	95% IC	*p*-Value	P adj
	<50 Years (N = 180) N (%)	≥50 Years (N = 100) N (%)					
T280M							
T/T	132 (73.3)	3 (3.0)	127.36	88.91	26.90–293.89	<0.001	<0.001
T/M	47 (26.1)	23 (23.0)					
M/M	1 (0.5)	74 (74.0)					
T	311 (86.4)	29 (13.2)	278.57	37.42	22.80–61.4	<0.001	<0.001
M	49 (13.6)	171 (86.8)					
V249I							
V/V	84 (46.7)	53 (53.0)	1.03	0.77	0.47–1.27	0.31	0.4
V/I	85 (47.2)	40 (40.0)					
I/I	11 (6.1)	7 (7.0)					
V	253 (70.3)	146 (73.0)	0.46	0.87	0.59–1.28	0.5	0.5
I	107 (29.7)	54 (27.0)					

Abbreviations: OR: Odds ratio; 95% IC: 95% confidence interval; N: total number of subjects. Significant *p*-value: *p*-value < 0.05. P adj: *p*-values adjusted using Benjamini–Hochberg FDR correction. *p*-value for genotype frequencies was obtained when comparing T/T genotype and T/M + M/M genotype for the nonsynonymous SNP T280M. *p*-value for genotype frequencies was obtained when comparing V/V genotype and V/I + I/I genotype for the nonsynonymous SNP V249I.

## Data Availability

The data that support the findings of this study are available from the corresponding author upon reasonable request.

## Abbreviations

The following abbreviations are used in this manuscript:

HBV	Hepatitis B Virus
CHB	Chronic hepatitis B virus
CX3CR1	Fractalkine-CX3CL1-Receptor
ICA	Internal Carotid Artery
EDTA	Ethylenediaminetetraacetic acid
PCR-RFLP	Polymerase Chain Reaction–Restriction Fragment Length Polymorphisms
OR	Odds Ratio
CI	Confidence Intervals
FDR	Benjamini–Hochberg False Discovery Rate
